# Vitamin E Attenuating Effects Against the Impact of the Herbicide Atrazine on the Diaphragm Muscle of Male Wistar Rats

**DOI:** 10.1155/jt/7995780

**Published:** 2025-03-05

**Authors:** Felipe Cantore Tiburcio, Viviane da Silva Martins Lopes Correa, Kevin Silva Muller, Ana Paula Silveira Leite, Carina Guidi Pinto, Fabio Anselmo, Antonio Francisco Godinho, Carlos Roberto Padovani, Ana Angelica Henrique Fernandes, Shelly Favorito de Carvalho, Selma Maria Michelin Matheus

**Affiliations:** ^1^Medical School, São Paulo State University (UNESP), Botucatu, São Paulo, Brazil; ^2^Division of Anatomy, Department of Structural and Functional Biology, Institute of Biosciences, São Paulo State University (UNESP), Botucatu, São Paulo, Brazil; ^3^Toxicological Information and Assistance Center (Ciatox), Institute of Biosciences, São Paulo State University (UNESP), Botucatu, São Paulo, Brazil; ^4^Division of Biostatistics, Department of Biodiversity and Biostatistics, Institute of Biosciences, São Paulo State University (UNESP), Botucatu, São Paulo, Brazil; ^5^Division of Chemistry and Biochemistry, Department of Chemical and Biological Sciences, Institute of Biosciences, São Paulo State University (UNESP), Botucatu, São Paulo, Brazil; ^6^Electron Microscopy Center, Institute of Biosciences, São Paulo State University (UNESP), Botucatu, São Paulo, Brazil

**Keywords:** antioxidant, atrazine, herbicide, neuromuscular junction, oxidative stress, pesticide, skeletal muscle, vitamin E

## Abstract

Atrazine is an herbicide associated with respiratory disorders and the presence of oxidative stress, which can be reversed by association with antioxidant compounds, such as vitamin E. This study aimed to investigate the impact of atrazine (AZ) on the male rat diaphragm muscle and the attenuating effects of vitamin E. Fifty-two male rats were received for 28 days by gavage (*n* = 13/group): C (control), corn oil; AZ (100 mg/kg); AZE, AZ (100 mg/kg) and vitamin E (200 mg/kg); E, vitamin E (200 mg/kg). Both oxidative stress analysis and morphological analysis of the diaphragm muscle, neuromuscular junction (NMJ), and phrenic nerve were performed. Exposure to AZ caused oxidative stress in muscle fibers, as evidenced by the highest lipid hydroperoxide, and hydrophilic antioxidant capacity values in the AZ group. However, in the AZE group, these values were like those of the C group. The area and diameter of the muscle fiber were only larger in the E group. Exposure to AZ caused oxidative stress in the diaphragm muscle, but vitamin E attenuated these alterations and protected muscle fibers from the oxidative damage. Therefore, vitamin E may serve as a useful attenuating agent against AZ-induced oxidative stress in the skeletal muscle.

## 1. Introduction

Pesticides are a critical component of modern agriculture, used to protect crops by preventing, controlling, or eradicating harmful organisms and diseases [1]. However, their use carries the risk of bioaccumulation in various organs and tissues, which can cause damage, biochemical alterations, and an increased metabolic burden associated with detoxification processes [2–4]. Pesticides can be classified by different pest targets, including fungicides, insecticides, herbicides, and rodenticides [5].

Atrazine (AZ) (2-chloro-4-ethylamino-6-isopropylamino-s-triazine) is an herbicide belonging to the group of s-triazines, accounting for 30% of world pesticide production of pesticides [6], widely used for weed control in corn, sorghum, sugar cane, pineapple, and cereals [7].

The correlation between pesticide exposure and the development of serious health problems and chronic diseases in humans is a growing concern. Specifically, AZ exposure has been linked to a variety of diseases, including cancer, neurological, dermatological, and respiratory disorders [7, 8]. Pulmonary alterations, such as asthma, wheezing, dyspnea, and chronic bronchitis, have been associated with occupational exposures to pesticides [9–11], but the harmfulness of environmental exposure remains unclear [9]. The Agricultural Health Study, one of the most important studies of respiratory pathologies, found a correlation between wheezing and AZ exposure, suggesting that this substance may be a problematic air pollutant [12], which has already been further demonstrated by experimental studies [13]. The environmental persistence of AZ is notable, with a degradation half-life ranging from approximately 95–350 days [7].

In several countries, aquatic systems close to farms have showed in their samples that the presence of AZ and its metabolites, which represent environmental pollution, is a significant threat to both the aquatic ecosystem and human health [4, 14–17].

AZ metabolism results in the formation of reactive oxygen species (ROS) [3, 18, 19] modulating the antioxidant defense system and causing oxidative damage [2, 20] to all cellular components, including nucleic acid, proteins, lipids, and even cellular death [21, 22]. The severe effects of pesticides are related to oxidative stress [23, 24], in which many studies have already demonstrated the direct effects of AZ in antioxidant enzymes, such as superoxide dismutase (SOD), catalase, lipid hydroperoxide (HP), and glutathione peroxidase directly interfering with the antioxidant defense mechanism [25–29], in both in vivo and in vitro models.

The diaphragm, a key muscle for breathing, plays a vital role in ensuring efficient respiration [30]. Dysfunction of the diaphragm, a common medical issue, can stem from various underlying health conditions, from myofiber weakness to atrophy and intoxication [30, 31]. Notably, lung disorders, particularly those involving parenchymal changes, can also lead to or result from compromised respiratory muscles [30]. Indeed, AZ has experimentally shown to induce structural modifications in the lungs, leading to an increase in mucus secretion [13]. Moreover, ATZ treatment has been found to cause structural alterations in the myocardium [32] and mitochondrial dysfunctions in muscle cell lines [26].

Vitamin E acts primarily as an antioxidant, neutralizing free radicals and protecting cell membranes from oxidative damage [33]. It can also regulate the production of ROS [20] by maintaining oxidative phosphorylation of the mitochondria [34]. Studies have highlighted the beneficial impact of combining vitamin E with AZ, demonstrating the amelioration of oxidative stress in various tissues and organs [35–37]. Notably, vitamin E has already shown to attenuate oxidative stress markers in muscle and nerve tissues [25, 38, 39] while also improving nerve myelination, blood flow, and growth after injury [40, 41] demonstrating potential for further attenuating effects.

Considering the increasing incidence of respiratory disorders related to exposure to AZ, there is an imperative need for research investigating the influence of AZ on respiratory skeletal muscles and neuromuscular interactions in mammals. This study aimed to evaluate the attenuating and antioxidant effects of vitamin E on nerves, muscle fibers, neuromuscular junctions (NMJs), and oxidative stress after exposure to AZ in the diaphragm muscle of rats.

## 2. Materials and Methods

### 2.1. Animals and Experimental Design

Fifty-two male Wistar rats (80 days old, 300–400 g) were used and kept in bioterium under appropriate conditions of temperature (22 ± 2°C), photoperiod (12:12 h), humidity, and ventilation, receiving water and food ad libitum. This study was approved by the Ethics Committee on Animal Use (CEUA) from Sao Paulo State University (Unesp), Institute of Botucatu Biosciences, protocol: 897/16.

The animals were divided into four groups (*n* = 13/group): control (C), AZ, AZ with vitamin E (AZE), and vitamin E (E). All animals received for 28 days by gavaging the substances according to experimental groups: C, 0.3 mL of corn oil vehicle; AZ (Nortox 500 SC) at a dose of 100 mg/kg; AZE received a mixture of AZ (100 mg/kg) and vitamin E (200 mg/kg; E, vitamin E diluted in corn oil vehicle (200 mg/kg).

The period and dose of AZ exposure were determined according to the protocol proposed by the Guidelines for the Testing of Chemicals and Pesticides of the Organization for Economic Cooperation and Development for subchronic, repeated oral toxicity studies in rodents [25]. The concentration of vitamin E used (200 mg/kg) was based on previous experimental studies, which used the same dose, and reported an antioxidant effect of vitamin E with a decrease in oxidative stress in various tissues of rats and other animals [42–45]. Furthermore, male rats were chosen due to the larger body of the literature and data relating to adverse respiratory issues about AZ exposure in male farmers [9, 10]. However, females are also affected, and these sex-dependent effects should be investigated.

After 28 days, the animals were weighed, anesthetized by intraperitoneal injection of Ketamine (Dopalen, 90 mg/kg) and Xylazine (Anasedan, 10 mg/kg), and sacrificed by decapitation. The diaphragm muscle fragments were dissected, processed, and subjected to the following analyses.

### 2.2. Oxidative Stress Assessment

Hundred mg of proximal portions of the diaphragm muscle were dissected from each animal, stored in cryotubes, and frozen in liquid nitrogen, for the following determinations. Glutathione peroxidase activity was determined according to the method of Zakowski and Tappel [46], where the enzyme was mixed with the sample in the presence of hydrogen peroxide, in which enzyme activity was measured by the disappearance of NADPH at 340 nm in a spectrophotometer; SOD activity was determined by the technique of Crouch et al. [47], based on the enzyme's ability to inhibit the reduction of nitroblue-tetrazolium by free radicals generated by hydroxylamine in an alkaline medium; catalase activity was determined using phosphate buffer pH 7.0, and0.5 mL of the sample incubated in the hydrogen peroxide (30%) solution. The readings were taken at 240 nm according to what was described by Aebi [48]; lipid HP was determined through the oxidation of ammoniacal ferrous sulfate in the presence of xylene orange, sulfuric acid, and butylated hydroxytoluene in methanol, at room temperature [29]; and hydrophilic antioxidant capacity (HAC) was determined fluorometrically, as described by Beretta et al. [49] using a VICTOR X2 reader (Perkin Elmer, Boston, MA). Briefly, the antioxidant capacity was quantified by comparing the area under the curve relative to the oxidation kinetics of the phosphatidylcholine suspension, which was used as a reference for the biological matrix. All analyses were performed in triplicate, and the results are presented as a percentage of protection.

### 2.3. Muscle Fiber Analysis

Diaphragm muscle lateral portions were dissected and formed into rolled tubes. These tubes were then flash-frozen in liquid nitrogen after immersion in neutral talc and stored at −80°C. Cryostat sectioning (6 μm, Leica CM 1800) yielded two slides per animal. One slide was stained with hematoxylin and eosin (H&E) and imaged using an Olympus BX41 microscope (20x magnification). Muscle fiber area and diameter were measured from approximately 200 fibers, selected from three to four random fields per slide, using ImageJ software (version 1.53) [50, 51].

### 2.4. Intramuscular Collagen Quantification

Intramuscular collagen quantification was performed using images of Picrosirius red-stained slides. This staining method differentially labeled collagen (red) and muscle fibers (yellow). Six randomly selected images per animal (20x magnification) were analyzed for each experimental group. The percentage of collagen was automatically calculated using Leica QWin software (Leica Microsystems) [50, 51].

### 2.5. Phrenic Nerve Analysis

A section of 1 cm of the right phrenic nerves, proximal to their insertion into the diaphragm muscle, was harvested, preserved in Karnovsky fixative (composed of 4% paraformaldehyde and 2.5% glutaraldehyde in 0.2 M phosphate buffer, pH 7.2), treated with 1% osmium tetroxide, embedded in Historesin, and cross sectioned using a microtome (with a thickness of 2 μm, model RM2265 by Leica). Subsequently, the slides were stained with Toluidine Blue [52]. These prepared slides were then subjected to morphometric analysis using ImageJ software (version 1.53), where the number, diameter, area of nerve fibers, diameter and area of axons, thickness of the myelin sheath, and G ratio were obtained [50, 52].

### 2.6. Neuromuscular Junction Analysis

The diaphragm muscle fragments containing the motor point were fixed in a Karnovsky fixative and sectioned longitudinally using a metal sheet. Slices were subjected to the nonspecific esterase reaction to mark the NMJs, as described by Muller et al. [53]. Images of 50 NMJ per animal for each experimental group were used for area and diameter analysis (ImageJ version 1.53) [50, 51, 53].

### 2.7. Acetylcholine Receptors in the Neuromuscular Junction Analysis

For acetylcholine receptors (AChR) visualization, diaphragm muscle samples (containing the motor point) were obtained from three animals per experimental group following transcardiac perfusion (PBS then 4% paraformaldehyde, 0.1 M phosphate-buffered saline, pH 7.4) and right auricle drainage. The labeling protocol involved: 15 min postfixation; 3x PBS washes (5 min, shaker); incubation in 0.1 M glycine (20 min, shaker); a PBS wash; incubation in 1% collagenase (Sigma C-0130, 20 min, shaker); a PBS wash; incubation in 4% Triton X-100 (Sigma T9284, in PBS, 1 h); and a final PBS wash (10 min). Rhodamine-conjugated alpha-bungarotoxin (Invitrogen/Thermo Fisher Scientific, T1175, 1 : 100 in PBS) was applied for 30 min at room temperature (shaker). Samples were then PBS washed, mounted in VECTASHIELD (Vector Laboratories), and imaged using a Leica TCS-5-SPE confocal microscope [51, 52, 54].

### 2.8. Statistical Analysis

The results of all quantitative variables were compared between the groups, and before any analysis, the normal distribution and homoscedasticity of the data were verified using the Kolmogorov–Smirnov test. Given that the comparison between the groups adhered to a normal probability distribution, all analyses were conducted using the variance analysis technique for the model with one factor (one-way ANOVA), complemented with Tukey's multiple comparisons tests, with a significance level of 5% (*p* < 0.05). The results were expressed as mean and standard deviation (SD), and the analyses were performed using the software “GraphPad Prism 9.” All graphs were created within the same software.

## 3. Results

### 3.1. Oxidative Stress Results

**Figure 1:**
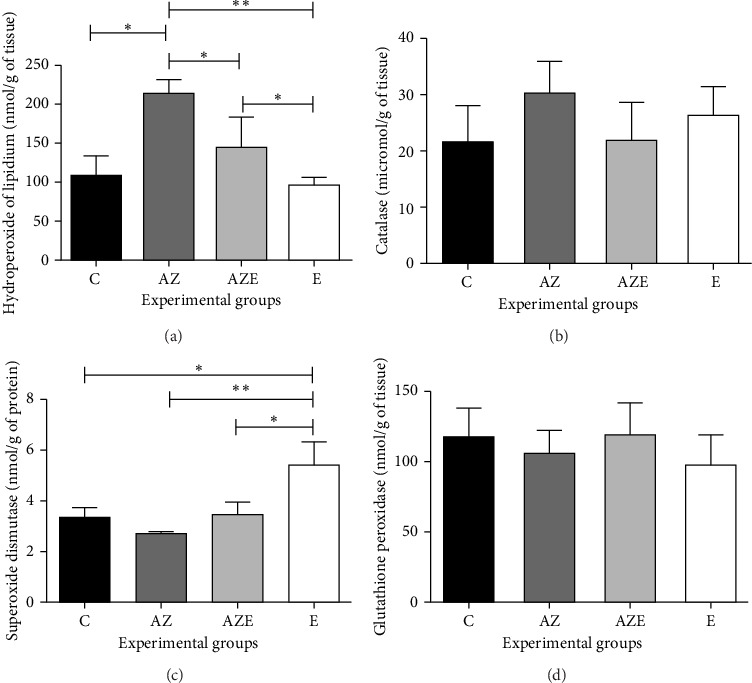
Graphs of the enzymatic determination of hydroperoxide of lipidium (a), catalase (b), superoxide dismutase (c), and glutathione peroxidase (d), according to the experimental groups (*n* = 7/group). Values expressed as means and standard deviation and analyzed by the one-way ANOVA test complemented by the Tukey multiple comparison test (⁣^∗^*p* < 0.05 and ⁣^∗∗^*p* < 0.01). C, control; AZ, atrazine; AZE, atrazine + vitamin E; E, vitamin E.

Regarding the activity of lipid HP (*F*_(3.20)_ = 27.35 (*p*=0.0001), the AZ group (214.29 ± 17.55) showed an increase compared to all groups: C (106.80 ± 24.80), E (94.81 ± 12.14), and AZE (144.92 ± 38.35). In the AZE when compared to AZ, there was a decrease in this parameter (*p* < 0.05). In terms of the E group (94.81 ± 12.14), there was a decrease in activity compared to AZE (144.92 ± 38.35) (Figure 1(a)).

The quantification of SOD (*F*_(3.20)_ = 28.96 (*p*=0.0001) revealed in the E (5.44 ± 0.89) group the highest values for this enzyme compared to all groups: C(3.40 ± 0.35), AZ (2.70 ± 0.11), and AZE (3.48 ± 0.47) (Figure 1(c)).

Analysis of the catalase (*F*_(3.20)_ = 2.77 (*p*=0.0682) and glutathione peroxidase enzyme (*F*_(3.20)_ = 1,39 [*p*=0.2755]) did not show differences between the experimental groups (Figures 1(b) and 1(d)).

**Figure 2:**
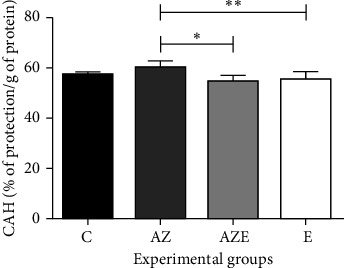
Graphs of hydrophilic antioxidant capacity (CAH) protection percentage, according to experimental groups (*n* = 7/group). Values expressed as means and standard deviation and analyzed by the one-way ANOVA test complemented by the Tukey multiple comparison test (⁣^∗^*p* < 0.05 and ⁣^∗∗^*p* < 0.01). C, control; AZ, atrazine; AZE, atrazine + vitamin E; E, vitamin E.

Data on the CAH (*F*_(3.20)_ = 7.14 (*p*=0.0019) evaluation are presented in percentage of protection. The results obtained showed the highest values in the AZ (60.24 ± 2.46) group compared to the AZE (54.42 ± 2.41) and E (55.32 ± 3.05). Between the C (57.29 ± 1.06) and the other groups, there was no significant differences (*p* > 0.05) (Figure 2).

### 3.2. Results of Muscle Fibers and Collagen Quantification

**Figure 3:**
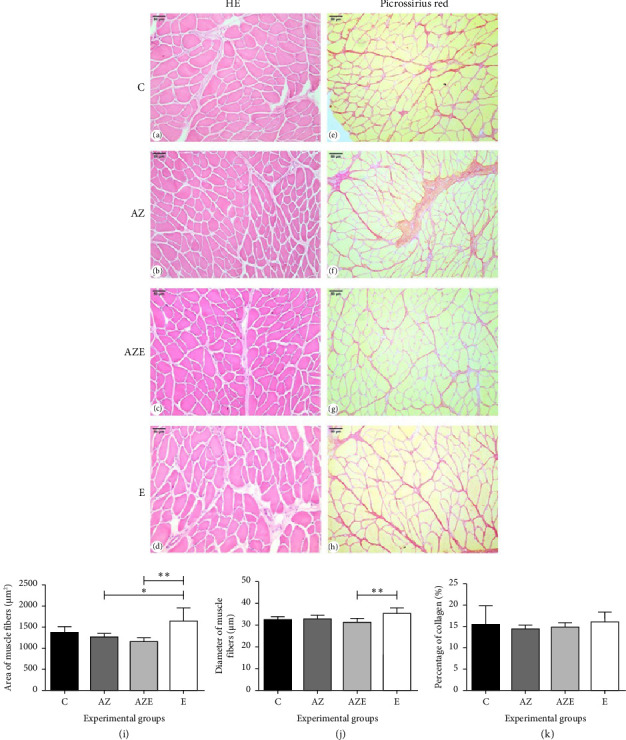
(a–h) Photomicrographs of diaphragm muscle cross sections stained with HE (a–d) and picrosirius red (e–h) of all experimental groups (*n* = 5/group), under conventional light microscopy with 200x magnification (scale bar = 50 μm). (i–k) Graphs of the area (i) and diameter (j) of muscle fiber and percentage of collagen (k) for all experimental groups. Values expressed as means and standard deviation and analyzed by the one-way ANOVA test complemented by the Tukey multiple comparison test (⁣^∗^*p* < 0.05 and ⁣^∗∗^*p* < 0.01). C, control; AZ, atrazine; AZE, atrazine + vitamin E; E, vitamin E.

Regarding the morphology of muscle fibers, there were no significant differences observed among the experimental groups. In all groups, the muscle fibers exhibited a polygonal shape with peripheral nuclei (Figures 3(a), 3(b), 3(c), and 3(d)). In addition, there was a moderate amount of extracellular matrix (collagen) present (Figures 3(e), 3(f), 3(g), and 3(h)), which is consistent with the normal characteristics.

The assessment of the muscle fiber area (*F*_(3.16)_ = 6.10 (*p*=0.0057) revealed in the E group (1639.46 ± 321.80) the highest values compared to AZ (1272.45 ± 82.58) and AZE (1145.74 ± 105.99) groups (Figure 3(i)). Regarding the diameter of muscle fibers (*F*_(3.16)_ = 4.29 (*p*=0.0211), the E group (35.46 ± 2.76) showed the highest values compared only to the AZE (31.13 ± 1.87) (Figure 3(j)). Collagen quantification (*F*_(3.16)_ = 0.46 (*p*=0.7129) (expressed as relative area percentage) did not reveal any statistically significant differences between the experimental groups (*p* > 0.05) (Figure 3(k)).

### 3.3. Phrenic Nerve Results

**Figure 4:**
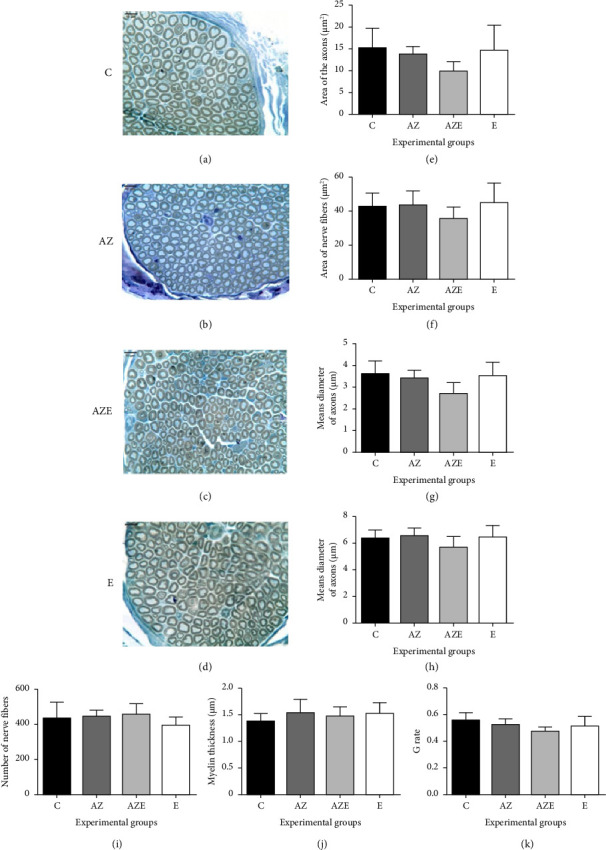
(a–d) Photomicrographs of phrenic nerve cross sections stained with osmium tetroxide and contrasted with toluidine blue, for all experimental groups (*n* = 5/group), under conventional light microscopy with 1000x magnification (scale bar = 10 μm). (e–k) Graphs of the area of axons (e), area of nerve fibers (f), mean diameter of axons (g), mean diameter of nerve fibers (h), number of nerve fibers (i), myelin sheath thickness (j), and the *G* ratio (k). Values expressed as means and standard deviation and analyzed by the one-way ANOVA test complemented by the Tukey multiple comparison test of multiple comparisons of Tukey (*p* < 0.05). C, control; AZ, atrazine; AZE, atrazine + vitamin E; E, vitamin E.

The morphological analysis revealed a consistent pattern of normality across all groups. Histologically, the axons exhibited an intact myelin sheath, showing no evidence of degeneration, endoneural edema, or inflammatory infiltration (Figures 4(a), 4(b), 4(c), and 4(d)). These findings were further validated by the morphometric analysis, which demonstrated no statistically significant differences among the groups of all analyzed parameters (*p* > 0.05) (Figures 4(e), 4(f), 4(g), 4(h), 4(i), 4(j), and 4(k)). Concerning the morphometric analysis, the values of F are, respectively, area of axons (*F*_(3.16)_ = 1.85 [*p*=0.1718]), area of nerve fibers (*F*_(3.16)_ = 1.15 [*p*=0.3596]), mean diameter of axons (*F*_(3.16)_ = 2.18 [*p*=0.1308]), mean diameter of nerve fibers (*F*_(3.16)_ = 1.75 [*p*=0.32607]), number of nerve fibers (*F*_(3.16)_ = 1.06 [*p*=0.3920]), myelin sheath thickness (*F*_(3.16)_ = 0.70 [*p*=0.5682]), and the G ratio (*F*_(3.16)_ = 2.36 [*p*=0.1104]).

### 3.4. NMJ Results

**Figure 5:**
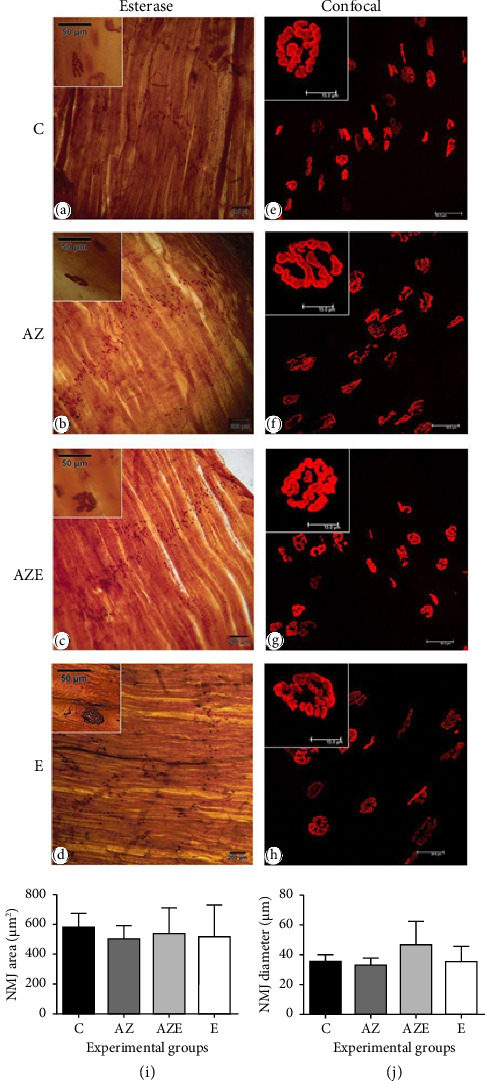
(a–h) Photomicrographs of the diaphragm muscle cross sections of all experimental groups (*n* = 5/group). (a–d) Neuromuscular junctions marked with nonspecific esterase reaction, under conventional light microscopy with 100x magnification (scale bar = 200 μm) or in detail with 400x magnification (scale bar = 50 μm). (e–h) Acetylcholine receptors of the neuromuscular junction marked with rhodamine-conjugated alpha-bungarotoxin, under laser scanning confocal microscopy with 400x magnification (scale bar = 50 μm) or in detail with 630x magnification (scale bar = 15 μm). Graphs of the area (i) and diameter (j) of the neuromuscular junction for all experimental groups. Values expressed as means and standard deviation and analyzed by the one-way ANOVA test complemented by the Tukey multiple comparison test of multiple comparisons of Tukey (*p* > 0.05). C, control; AZ, atrazine; AZE, atrazine + vitamin E; E, vitamin E.

The NMJ in all animals exhibited a transverse or oblique alignment with the major axis of muscle fibers, which is considered normal (Figures 5(a), 5(b), 5(c), and 5(d)), and no significant morphological alterations were observed (Figures 5(e), 5(f), 5(g), and 5(h)).

Regarding NMJ morphometry, the NMJ area (*F*_(3.16)_ = 0.70 [*p*=0.8924]) and diameter (*F*_(3.16)_ = 1.83 [*p*=0.1830]) did not show any statistical differences between the experimental groups (*p* > 0.05) (Figures 5(i) and 5(j)). In addition, the AChR at the NMJ demonstrated a distribution consistent with normality across all groups studied. The fluorophore responses were homogeneous, with AChR appearing in autofluorescence and forming continuous branches or “pretzel” shapes, extending along various orientations within the muscle fibers (Figures 5(e), 5(f), 5(g), and 5(h)).

## 4. Discussion

The metabolism of pesticides, including AZ, results in the formation of ROS [3, 18, 19]. This process modulates the antioxidant defense system, causing oxidative damage to all cellular components, including nucleic acids, lipids, and proteins [21, 22]. The toxicity generated promotes an increase in energy expenditure in the affected tissues, with the aim of their elimination. This demand may lead to protein degradation and amino acid production, thereby increasing adenosine triphosphate synthesis needed to maintain the tricarboxylic acid cycle [55].

Metabolic changes in tissues are accompanied by the generation of free radicals. The molecular mechanism involved in the generation of these radicals by AZ is not known. The reports available in plant studies suggest that AZ-induced toxicity may be related to inefficient activation of the oxygen removal pathways resulting in their accumulation, increases hydrogen peroxide (H_2_O_2_) content, reduces gene expression and enzyme activities related to two major H_2_O_2_ detoxification pathways, and induces the expression of a series of genes that encode oxidases [56].

The results of this study collaborate with these descriptions, allowing us to infer that AZ, at the dose used, caused oxidative stress in the diaphragm muscle of rats, as higher values were obtained in the AZ group concerning HP and CAH. Furthermore, vitamin E attenuated this response, as AZE was similar to C, but both differed from AZ regarding those parameters. The association with vitamin E has lowered AZ effects, although the vitamin E alone group demonstrated a higher capacity for this antioxidative potential, as the E group differed from AZE for SOD and HP activity. All those determinations provide a deeper understanding of the role of oxidative stress in various pathophysiological conditions and allow for inference about the effectiveness of antioxidant interventions [57]. A reduction in antioxidant enzyme activity has also been observed in several pesticide studies [27, 58–61].

Long-term exposure of cells to ROS leads to a reduction in natural endogenous antioxidants such as SOD [62]. This is consistent with the lower SOD values observed in Group AZ and the higher values found in Group E. On the other hand, the opposite performance was observed in the HP values, higher in the Z group and lower in the E group.

These results suggest an antioxidant action of vitamin E. The role of vitamin E is known in animal health for inactivating free radicals produced as products of normal cell activity, as well as for avoiding the formation of various stressors [58].

The literature reports that a vitamin-rich diet can help prevent several diseases [63, 64]. This is due to the synergistic action of exogenous and endogenous antioxidants in combating free radicals [65].

The SOD enzyme catalyzes the dismutation of the superoxide radical into H_2_O_2_, which can then be converted into water by CAT or glutathione peroxidase [66]. The similarity of the values found between all groups about glutathione peroxidase can be justified by the fact that CAT played an important role in the oxidative stress process, which also has the function of reducing hydrogen peroxide in water [66, 67], not requiring the action of glutathione.

Several authors have described the changes resulting from AZ in the central nervous system [68–70], but little has been studied on the action of this herbicide in the peripheral nervous system.

Respiratory complications are described as the result of the action of pesticides, including AZ [70, 71]. Although our study did not perform functional analyses associated with respiratory capacity, both morphological and morphometric results indicate that the phrenic nerve was preserved. The G ratio is a parameter associated with the conduction speed of nerve impulses, in which low G ratio values (around 0.4) typically correlate with axonal degeneration, while high G ratio values (around 0.7) suggest myelin degeneration or regeneration [72]. In this study, the obtained G ratio values across all groups centered around 0.52, confirming patterns of normality.

Given the relevance of the diaphragm for respiration, its dysfunction, which can be manifested through alterations in its fiber's composition or NMJ's organization, directly impacts respiratory issues [30, 31]. Here, no major morphological or morphometric changes due to AZ were found in muscle fibers and NMJs, as well as in the percentage of collagen. However, although no significant differences were observed between the studied groups in both morphology and morphometry for neuromuscular structures, the association with vitamin E increased the diameter of muscle fibers.

In our study, the results observed in the group that received only vitamin E indicated an increase in the area and a proportional increase in the diameter of muscle fibers. Its association with AZ promoted a decrease in these values, but these were equivalent to those of the control group. Indeed, vitamin E benefits myoblast proliferation, differentiation, and survival, while also acting on membrane repair, mitochondrial efficiency, mass, and contractile muscle properties, and the ability to exercise [73, 74]. These results can be associated with increased muscle effectiveness, as vitamin E has already shown to enhance muscle functions [74].

Vitamin E is also considered to decrease protein degradation and increase its synthesis [75]. The studies of Bartali et al. [76] and Chung et al. [74] described that vitamin E supplementation improves muscle functions, attenuating functional decline during aging, potentially due to its suppressive action on oxidative stress and inflammation. However, the effects of vitamin E on the muscle structure may not be explained exclusively by the elimination of free radicals. These effects may reflect specific interactions of alpha-tocopherol with enzymes, structural proteins, lipids, and transcription factors [77].

Eidi et al. [78] studying memory retention in rats evaluated the association of vitamin E with nicotine and pilocarpine. They found that vitamin E improved the responses of these drugs, suggesting that vitamin E and the cholinergic system have a close interaction. Therefore, the toxicity of pesticides should be increasingly considered, taking into account their conformations, molecular coupling, and the interactions that result from their molecular dynamics [79].

On the other hand, vitamin E, in addition to its antioxidant effect, interacts specifically with enzymes, structural proteins, lipids, and transcription factors and can be considered a potentially important agent in the prevention and treatment of cardiovascular diseases, cancer, inflammation, immunological, and neurodegenerative disorders [80]. It attenuates ROS by reducing oxygen, blocking enzymes involved in ROS generation (lipoxygenase, cycloxygenase, xanthine oxidase, and monooxygenase), chelating transition metal ions that trigger ROS production and eliminate lipid peroxidation, and helping in the recycling of other antioxidants [81].

Our study is innovative in its consideration of the effect of AZ on neuromuscular interactions, although no toxicity effects were found from subchronic exposure to AZ in the experimental model used. However, there is the lack of a functional analysis of the diaphragm muscle, related to respiratory function, and also no analysis of female rats because this would require control of the estrous cycle. Further studies including those parameters may be interesting to improve our current knowledge and guarantee that those neuromuscular structures are not hindered after subchronic exposure to AZ.

Considering that AZ is a stable structured pest infestation, difficult to degrade, and that it remains for a long time in the environment [82], whose toxicity occurs in the long term [24], our study contributes to new nutritional strategies associated with the use of vitamin E to ameliorate adverse effects to mammals. Furthermore, interest in the beneficial functionalities of this vitamin is increasing and will continue to attract researchers.

## 5. Conclusions

This study demonstrates that exposure to AZ, at the administered dose, induces oxidative stress in the fibers of the rat diaphragm muscle. However, despite AZ's pro-oxidant effect in the experimental model used, it did not result in significant morphological changes in the neuromuscular interaction (muscle fibers, nerves, and NMJs). A key finding is that vitamin E reverses this oxidative stress and protects muscle fibers from oxidative damage, suggesting its potential as an effective antioxidant, which attenuates AZ-induced oxidative stress in the skeletal muscle. These findings have substantial implications for understanding the effects of AZ on muscle health and underscore the therapeutic potential of vitamin E [83].

## Data Availability

The datasets used and analyzed during the study are available without restriction upon request.

## References

[1] Béranger R., Hardy E. M., Binter A.-C. (2020). Multiple Pesticides in Mothers’ Hair Samples and Children’s Measurements at Birth: Results From the French National Birth Cohort (ELFE). *International Journal of Hygiene and Environmental Health*.

[2] Dornelles M. F., Oliveira G. T. (2016). Toxicity of Atrazine, Glyphosate, and Quinclorac in Bullfrog Tadpoles Exposed to Concentrations Below Legal Limits. *Environmental Science and Pollution Research*.

[3] Liu L., Li M.-Z., Yao M.-H., Yang T.-N., Tang Y.-X., Li J.-L. (2024). Melatonin Inhibits Atrazine-Induced Mitochondrial Impairment in Cerebellum of Mice: Modulation of cGAS-STING-NLRP3 Axis-Dependent Cell Pyroptosis. *Science of the Total Environment*.

[4] Li R., Hu W., Liu H. (2024). Occurrence, Distribution and Ecological Risk Assessment of Herbicide Residues in Cropland Soils From the Mollisols Region of Northeast China. *Journal of Hazardous Materials*.

[5] Tudi M., Daniel Ruan H., Wang L. (2021). Agriculture Development, Pesticide Application and its Impact on the Environment. *International Journal of Environmental Research and Public Health*.

[6] Abarikwu S. O., Farombi E. O. (2015). Atrazine Induces Apoptosis of SH-Sy5y Human Neuroblastoma Cells via the Regulation of Bax/Bcl-2 Ratio and Caspase-3-Dependent Pathway. *Pesticide Biochemistry and Physiology*.

[7] Jestadi D. B., Phaniendra A., Babji U., Srinu T., Shanmuganathan B., Periyasamy L. (2014). Effects of Short Term Exposure of Atrazine on the Liver and Kidney of Normal and Diabetic Rats. *Journal of Toxicology*.

[8] Rathinam X., Kota R., Thiyagar N. (2005). Farmers and Formulations—Rural Health Perspective. *Medical Journal of Malaysia*.

[9] Mamane A., Raherison C., Tessier J.-F., Baldi I., Bouvier G. (2015). Environmental Exposure to Pesticides and Respiratory Health. *European Respiratory Review*.

[10] Mamane A., Baldi I., Tessier J.-F., Raherison C., Bouvier G. (2015). Occupational Exposure to Pesticides and Respiratory Health. *European Respiratory Review*.

[11] Mostafalou S., Abdollahi M. (2017). Pesticides: An Update of Human Exposure and Toxicity. *Archives of Toxicology*.

[12] Hoppin J. A., Umbach D. M., Long S. (2014). Respiratory Disease in United States Farmers. *Occupational and Environmental Medicine*.

[13] D’Amico D., Fiore R., Caporossi D. (2021). Function and Fiber-Type Specific Distribution of Hsp60 and αB-Crystallin in Skeletal Muscles: Role of Physical Exercise. *Biology*.

[14] Ji Y., Dong C., Kong D., Lu J., Zhou Q. (2015). Heat-Activated Persulfate Oxidation of Atrazine: Implications for Remediation of Groundwater Contaminated by Herbicides. *Chemical Engineering Journal*.

[15] Liu Z., Wang Y., Zhu Z. (2016). Atrazine and its Main Metabolites Alter the Locomotor Activity of Larval Zebrafish (Danio Rerio). *Chemosphere*.

[16] Cai Y., Tian T., Huang Y. (2023). Occurrence and Health Risks of Organic Micropollutants in Tap Water in Dalian. *Chemical Research in Toxicology*.

[17] Hook S. E., Smith R. A., Waltham N., Warne M. S., St J. W. (2024). Pesticides in the Great Barrier Reef Catchment Area: Plausible Risks to Fish Populations. *Integrated Environmental Assessment and Management*.

[18] Figueira F. H., de Aguiar L. M., Rosa C. E. (2017). Embryo-Larval Exposure to Atrazine Reduces Viability and Alters Oxidative Stress Parameters in *Drosophila melanogaster*. *Comparative Biochemistry and Physiology-Part C: Toxicology & Pharmacology*.

[19] Yoon D.-S., Park J. C., Park H. G., Lee J.-S., Han J. (2019). Effects of Atrazine on Life Parameters, Oxidative Stress, and Ecdysteroid Biosynthetic Pathway in the Marine Copepod Tigriopus Japonicus. *Aquatic Toxicology*.

[20] Lin J., Zhao H.-S., Qin L. (2018). Atrazine Triggers Mitochondrial Dysfunction and Oxidative Stress in Quail (Coturnix C. Coturnix) Cerebrum via Activating Xenobiotic-Sensing Nuclear Receptors and Modulating Cytochrome P450 Systems. *Journal of Agricultural and Food Chemistry*.

[21] Pogrmic-Majkic K., Fa S., Dakic V., Kaisarevic S., Kovacevic R. (2010). Upregulation of Peripubertal Rat Leydig Cell Steroidogenesis Following 24 H In Vitro and In Vivo Exposure to Atrazine. *Toxicological Sciences*.

[22] Keshk W. A., Soliman N. A., Abo El-Noor M. M., Wahdan A. A., Shareef M. M. (2014). Modulatory Effects of Curcumin on Redox Status, Mitochondrial Function, and Caspace-3 Expression During Atrazin-Induced Toxicity. *Journal of Biochemical and Molecular Toxicology*.

[23] Semren T. Ž., Žunec S., Pizent A. (2018). Oxidative Stress in Triazine Pesticide Toxicity: A Review of the Main Biomarker Findings. *Archives of Industrial Hygiene and Toxicology*.

[24] Sánchez O. F., Lin L., Bryan C. J., Xie J., Freeman J. L., Yuan C. (2020). Profiling Epigenetic Changes in Human Cell Line Induced by Atrazine Exposure. *Environmental Pollution*.

[25] Ryan M. J., Dudash H. J., Docherty M. (2010). Vitamin E and C Supplementation Reduces Oxidative Stress, Improves Antioxidant Enzymes and Positive Muscle Work in Chronically Loaded Muscles of Aged Rats. *Experimental Gerontology*.

[26] Kapley A., Sagarkar S., Gandhi D., Devi S., Sakharkar A. (2016). Atrazine Exposure Causes Mitochondrial Toxicity in Liver and Muscle Cell Lines. *Indian Journal of Pharmacology*.

[27] Singh S., Kumar V., Chauhan A. (2018). Toxicity, Degradation and Analysis of the Herbicide Atrazine. *Environmental Chemistry Letters*.

[28] Das S., Sakr H., Al-Huseini I. (2023). Atrazine Toxicity: The Possible Role of Natural Products for Effective Treatment. *Plants*.

[29] Qian H., Zhao H., Ye H. (2024). Toxic Effects of Atrazine on Liver and Underlying Mechanism: A Review. *Expo Health*.

[30] Supinski G. S., Morris P. E., Dhar S., Callahan L. A. (2018). Diaphragm Dysfunction in Critical Illness. *Chest*.

[31] Manders E., de Man F. S., Handoko M. L. (2012). Diaphragm Weakness in Pulmonary Arterial Hypertension: Role of Sarcomeric Dysfunction. *American Journal of Physiology-Lung Cellular and Molecular Physiology*.

[32] Rajkovic V., Kovac R., Koledin I., Matavulj M. (2014). Atrazine-Induced Changes in the Myocardial Structure of Peripubertal Rats. *Toxicology and Industrial Health*.

[33] Arjmand K., Daneshi E., Pourmasumi S., Fathi F., Nasseri S., Sabeti P. (2023). Evaluation of the Effect of Vitamin E on Reproductive Parameters in Morphine-Treated Male Mice. *Addict Health*.

[34] Punz A., Nanobashvili J., Fuegl A., Huk I., Roth E. (1998). Effect of α-Tocopherol Pretreatment on High Energy Metabolites in Rabbit Skeletal Muscle After Ischemiareperfusion. *Clinical Nutrition*.

[35] Mansour S., Abdel-Mageed M., Mohamed K., Gad M., Gamet-Payrastre L. (2017). Adverse Effects to Suckling Mice Following Indirect Exposure to a Pesticide Mixture and Ameliorative Effect of α-Tocopherol Coadministration. *Journal of Basic and Clinical Health Sciences*.

[36] Erdemli M. E., Zayman E., Erdemli Z., Gul M., Gul S., Gozukara Bag H. (2020). Protective Effects of Melatonin and Vitamin E in Acetamiprid-Induced Nephrotoxicity. *Environmental Science and Pollution Research*.

[37] Abouelghar G. E., El-Bermawy Z. A., Salman H. M. S. (2020). Oxidative Stress, Hematological and Biochemical Alterations Induced by Sub-Acute Exposure to Fipronil (COACH®) in Albino Mice and Ameliorative Effect of Selenium Plus Vitamin E. *Environmental Science and Pollution Research*.

[38] Menorca R. M. G., Fussell T. S., Elfar J. C. (2013). Peripheral Nerve Trauma: Mechanisms of Injury and Recovery. *Hand Clinics*.

[39] Riffel A. P. K., Santos M. C. Q., de Souza J. A. (2018). Treatment With Ascorbic Acid and α-Tocopherol Modulates Oxidative-Stress Markers in the Spinal Cord of Rats with Neuropathic Pain. *Brazilian Journal of Medical and Biological Research*.

[40] Albay C., Akkalp A. K. (2021). Alpha-Tocopherol and Cyanocobalamin Combination Accelerates Peripheral Nerve Healing: An Experimental Animal Study. *Turk Neurosurg*.

[41] Ölke H. C., Biçer Ö.S., Mirioğlu A., Şaker D., Öcal I., Özkan C. (2023). Clinical, Electrophysiological, and Histomorphological Effects of Local Coenzyme Q10 and Vitamin E Use in a Rat Model of Peripheral Nerve Injury. *Acta Orthopaedica et Traumatologica Turcica*.

[42] Jensen C., Flensted-Jensen M., Skibsted L. H., Bertelsen G. (1998). Effects of Dietary Rape Seed Oil, Copper(II) Sulphate and Vitamin E on Drip Loss, Colour and Lipid Oxidation of Chilled Pork Chops Packed in Atmospheric Air or in a High Oxygen Atmosphere. *Meat Science*.

[43] Kalender Y., Uzunhisarcikli M., Ogutcu A., Acikgoz F., Kalender S. (2006). Effects of Diazinon on Pseudocholinesterase Activity and Haematological Indices in Rats: The Protective Role of Vitamin E. *Environmental Toxicology and Pharmacology*.

[44] Boler D. D., Gabriel S. R., Yang H. (2009). Effect of Different Dietary Levels of Natural-Source Vitamin E in Grow-Finish Pigs on Pork Quality and Shelf Life. *Meat Science*.

[45] Çoban J., Bingül I., Yesil-Mizrak K., Dogru-Abbasoglu S., Oztezcan S., Uysal M. (2013). Effects of Carnosine Plus Vitamin E and Betaine Treatments on Oxidative Stress in Some Tissues of Aged Rats. *Current Aging Science*.

[46] Zakowski J. J., Tappel A. L. (1978). Purification and Properties of Rat Liver Mitochondrial Glutathione Peroxidase. *Biochimica et Biophysica Acta (BBA)-Enzymology*.

[47] Crouch R. K., Gandy S. E., Kimsey G., Galbraith R. A., Galbraith G. M., Buse M. G. (1981). The Inhibition of Islet Superoxide Dismutase by Diabetogenic Drugs. *Diabetes*.

[48] Aebi H. (1984). Catalase In Vitro. *Methods in Enzymology*.

[49] Beretta G., Aldini G., Facino R. M., Russell R. M., Krinsky N. I., Yeum K.-J. (2006). Total Antioxidant Performance: A Validated Fluorescence Assay for the Measurement of Plasma Oxidizability. *Analytical Biochemistry*.

[50] Leite A. P. S., Pinto C. G., Tibúrcio F. C. (2019). Heterologous Fibrin Sealant Potentiates Axonal Regeneration after Peripheral Nerve Injury With Reduction in the Number of Suture Points. *Injury*.

[51] Tibúrcio F. C., Leite A. P. S., Muller K. S. (2023). Effects of Nandrolone Decanoate on Skeletal Muscle and Neuromuscular Junction of Sedentary and Exercised Rats. *Medicina (Kaunas)*.

[52] Leite A. P. S., Pinto C. G., Tibúrcio F. C. (2023). Acetylcholine Receptors of the Neuromuscular Junctions Present Normal Distribution After Peripheral Nerve Injury and Repair Through Nerve Guidance Associated With Fibrin Biopolymer. *Injury*.

[53] Muller K. S., Tibúrcio F. C., de Barros J. W. F., Matsumura C. Y., Matheus S. M. M. (2023). Statin Exposure During Pregnancy Promotes Neuromuscular Junction Alterations in Postpartum Wistar Rats. *Muscle & Nerve*.

[54] Pinto C. G., Leite A. P. S., Sartori A. A. (2021). Heterologous Fibrin Biopolymer Associated to a Single Suture Stitch Enables the Return of Neuromuscular Junction to its Mature Pattern After Peripheral Nerve Injury. *Injury*.

[55] Ganeshwade R. B. D., Deshmukh D., Sonawane S., Ashwini G. (2012). Toxicity of Endosulfan on Freshwater Fish Channa Striatus. *Trends in fisheries research*.

[56] Ramel F., Sulmon C., Bogard M., Couée I., Gouesbet G. (2009). Differential Patterns of Reactive Oxygen Species and Antioxidative Mechanisms During Atrazine Injury and Sucrose-Induced Tolerance in Arabidopsis Thaliana Plantlets. *BMC Plant Biology*.

[57] Fraga C. G., Oteiza P. I., Galleano M. (2014). In Vitro Measurements and Interpretation of Total Antioxidant Capacity. *Biochimica et Biophysica Acta (BBA)-General Subjects*.

[58] Chaâbane M., Elwej A., Ghorbel I. (2018). Penconazole Alters Redox Status, Cholinergic Function and Lung’s Histoarchitecture of Adult Rats: Reversal Effect of Vitamin E. *Biomedicine & Pharmacotherapy*.

[59] Griboff J., Morales D., Bertrand L. (2014). Oxidative Stress Response Induced by Atrazine in Palaemonetes Argentinus: The Protective Effect of Vitamin E. *Ecotoxicology and Environmental Safety*.

[60] Hassani S., Sepand M. R., Jafari A. (2015). Protective Effects of Curcumin and Vitamin E against Chlorpyrifos-Induced Lung Oxidative Damage. *Human & Experimental Toxicology*.

[61] Liu W., Du Y., Liu J. (2014). Effects of Atrazine on the Oxidative Damage of Kidney in Wister Rats. *International Journal of Clinical and Experimental Medicine*.

[62] Di Vincenzo A., Tana C., El Hadi H., Pagano C., Vettor R., Rossato M. (2019). Antioxidant, Anti-Inflammatory, and Metabolic Properties of Tocopherols and Tocotrienols: Clinical Implications for Vitamin E Supplementation in Diabetic Kidney Disease. *International Journal of Molecular Sciences*.

[63] Fiedor J., Burda K. (2014). Potential Role of Carotenoids as Antioxidants in Human Health and Disease. *Nutrients*.

[64] Mangge H., Becker K., Fuchs D., Gostner J. M. (2014). Antioxidants, Inflammation and Cardiovascular Disease. *World Journal of Cardiology*.

[65] Montezano A. C., Touyz R. M. (2014). Reactive Oxygen Species, Vascular Noxs, and Hypertension: Focus on Translational and Clinical Research. *Antioxidants and Redox Signaling*.

[66] Wang Y., Branicky R., Noë A., Hekimi S. (2018). Superoxide Dismutases: Dual Roles in Controlling ROS Damage and Regulating ROS Signaling. *Journal of Cell Biology*.

[67] Olson K. R., Gao Y., DeLeon E. R. (2017). Catalase as a Sulfide-Sulfur Oxido-Reductase: An Ancient (And Modern?) Regulator of Reactive Sulfur Species (RSS). *Redox Biology*.

[68] Kale O. E., Oyesola T. O., Raji F. S. (2018). Celecoxib, A Cyclooxygenase-2 Inhibitor, Offers Chemoprevention Against Reproductive and Neurobehavioural Abnormalities Induced by Atrazine in Male Wistar Rats. *Environmental Toxicology and Pharmacology*.

[69] Zhang B., Ma K., Li B. (2015). Inflammatory Reaction Regulated by Microglia Plays a Role in Atrazine-Induced Dopaminergic Neuron Degeneration in the Substantia Nigra. *Journal of Toxicological Sciences*.

[70] Eddleston M., Mohamed F., Davies J. O. J. (2006). Respiratory Failure in Acute Organophosphorus Pesticide Self-Poisoning. *QJM: International Journal of Medicine*.

[71] Bird S. B., Krajacic P., Sawamoto K., Bunya N., Loro E., Khurana T. S. (2016). Pharmacotherapy to Protect the Neuromuscular Junction After Acute Organophosphorus Pesticide Poisoning. *Annals of the New York Academy of Sciences*.

[72] Mendonça A. C., Barbieri C. H., Mazzer N. (2003). Directly Applied Low Intensity Direct Electric Current Enhances Peripheral Nerve Regeneration in Rats. *Journal of Neuroscience Methods*.

[73] von Grabowiecki Y., Licona C., Palamiuc L. (2015). Regulation of a Notch3-Hes1 Pathway and Protective Effect by a Tocopherol-Omega Alkanol Chain Derivative in Muscle Atrophy. *Journal of Pharmacology and Experimental Therapeutics*.

[74] Chung E., Mo H., Wang S. (2018). Potential Roles of Vitamin E in Age-Related Changes in Skeletal Muscle Health. *Nutrition Research*.

[75] Servais S., Letexier D., Favier R., Duchamp C., Desplanches D. (2007). Prevention of Unloading-Induced Atrophy by Vitamin E Supplementation: Links Between Oxidative Stress and Soleus Muscle Proteolysis?. *Free Radical Biology and Medicine*.

[76] Bartali B., Frongillo E. A., Guralnik J. M. (2008). Serum Micronutrient Concentrations and Decline in Physical Function Among Older Persons. *JAMA*.

[77] Hasan M. Y., Alshuaib W. B., Adem A., Singh S., Fahim M. A. (2004). α-Tocopherol Modifies Lead Induced Functional Changes at Murine Neuromuscular Junction. *Free Radical Research*.

[78] Eidi A., Eidi M., Mahmoodi G., Oryan S. (2006). Effect of Vitamin E on Memory Retention in Rats: Possible Involvement of Cholinergic System. *European Neuropsychopharmacology*.

[79] Mladenović M., Arsić B. B., Stanković N. (2018). The Targeted Pesticides as Acetylcholinesterase Inhibitors: Comprehensive Cross-Organism Molecular Modelling Studies Performed to Anticipate the Pharmacology of Harmfulness to Humans In Vitro. *Molecules*.

[80] Szymańska R., Nowicka B., Kruk J. (2017). Vitamin E-Occurrence, Biosynthesis by Plants and Functions in Human Nutrition. *Mini-Reviews in Medicinal Chemistry*.

[81] Mierziak J., Kostyn K., Kulma A. (2014). Flavonoids as Important Molecules of Plant Interactions With the Environment. *Molecules*.

[82] He H., Liu Y., You S., Liu J., Xiao H., Tu Z. (2019). A Review on Recent Treatment Technology for Herbicide Atrazine in Contaminated Environment. *International Journal of Environmental Research and Public Health*.

[83] Tintignac L., Brenner H., Rüegg M. (2015). Mechanisms Regulating Neuromuscular Junction Development and Function and Causes of Muscle Wasting. *Physiological Reviews*.

